# Tracing the developmental origin of tissue stem cells

**DOI:** 10.1111/dgd.12816

**Published:** 2022-11-01

**Authors:** Ritsuko Morita, Hironobu Fujiwara

**Affiliations:** ^1^ Laboratory for Tissue Microenvironment RIKEN Center for Biosystems Dynamics Research (BDR) Kobe Japan

**Keywords:** lineage tracing, origin of tissue stem cells, stem cell plasticity, telescope model, tissue topology

## Abstract

Tissue stem cells are vital for organ homeostasis and regeneration owing to their ability to self‐renew and differentiate into the various cell types that constitute organ tissue. These stem cells are formed during complex and dynamic organ development, necessitating spatial–temporal coordination of morphogenetic events and cell fate specification during this process. In recent years, technological advances have enabled the tracing of the cellular dynamics, states, and lineages of individual cells over time in relation to tissue morphological changes. These dynamic data have not only revealed the origin of tissue stem cells in various organs but have also led to an understanding of the molecular, cellular, and biophysical bases of tissue stem cell formation. Herein, we summarize recent findings on the developmental origin of tissue stem cells in the hair follicles, intestines, brain, skeletal muscles, and hematopoietic system, and further discuss how stem cell fate specification is coordinated with tissue topology.

## Graphical abstract

This review focus on how tissue stem cells are formed during organ development. We introduce recent findings on the developmental origin of tissue stem cells in various organs and further discuss the coordination of stem cell fate specification and tissue topology that recent emerging technologies have revealed.

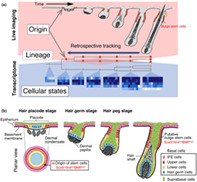

## 1 INTRODUCTION—UNANSWERED QUESTIONS IN TISSUE STEM CELL RESEARCH

The human body loses billions of cells daily through tissue turnover and damage. These cells are replenished through the actions of adult tissue stem cells, which serve as a source of specialized cells owing to their long‐term self‐renewal capacity and ability to generate differentiated cells within the tissue (Xin et al., 2016). Adult tissue stem cells were first discovered in the hematopoietic system in the 1960s (Moore & Owen, 1967; Till & McCulloch, 1961). Since then, a wide variety of adult tissue stem cells have been identified in most adult tissues and organs, such as the bone marrow, muscle, skin, intestines, and brain (Saba et al., 2021; Xin et al., 2016). Additionally, technological advances in molecular biology and genetics have facilitated the identification of genes that are specifically expressed in stem cells. These advances have also enabled the labeling, visualization, isolation, and manipulation of stem cells in vivo and in vitro. This has facilitated a greater understanding of stem cell gene expression profiles, the molecular and cellular entities of stem cell niches, and the mechanisms that regulate stem cell proliferation and differentiation via stem cell–niche interactions (Saba et al., 2021; Xin et al., 2016).

Conversely, while definitive tissue stem cells are believed to emerge from embryonic progenitor cells in tissue as the product of a hierarchy of decisions controlled by inducing factors (Slack, 2008), the developmental origin of tissue stem cells, as well as the molecular and cellular mechanisms underlying the emergence of tissue stem cells, is largely unknown. This leaves a knowledge gap regarding where tissue stem cells originate from and whether adult tissue stem cells emerge through maintaining their fetal undifferentiated state (default state) or through induction. It is also unclear how tissue stem cells are formed in the correct place and time relative to complex organ morphogenetic events and how the interdependent relationship between stem cells and niches is established. Moreover, it is unknown whether a common stem/progenitor cell population drives developmental and adult organ formation. Knowledge of these processes is crucial, not only to facilitate an understanding of the basic principles of developmental biology but also to decipher the design principles of organ homeostasis in an adult context. Recent advances in genetic engineering, imaging techniques, and theoretical biology have provided new insights into the origin and formation processes of tissue stem cells.

In this review, we summarize the existing knowledge regarding the developmental origins of tissue stem cells in various organs, which has been enabled by advances in genetic lineage tracing. We then discuss recent findings regarding the developmental origin of mouse hair follicle stem cells through a nonbiased, comprehensive lineage tracing strategy—a combination of long‐term live imaging and single‐cell transcriptomics. Finally, we discuss the contribution of the formation of organ‐specific tissue architecture and patterning in the emergence of tissue stem cells using the mammalian intestine as an example.

## 2 ELUCIDATING THE DEVELOPMENTAL ORIGIN OF TISSUE STEM CELLS

**FIGURE 1:**
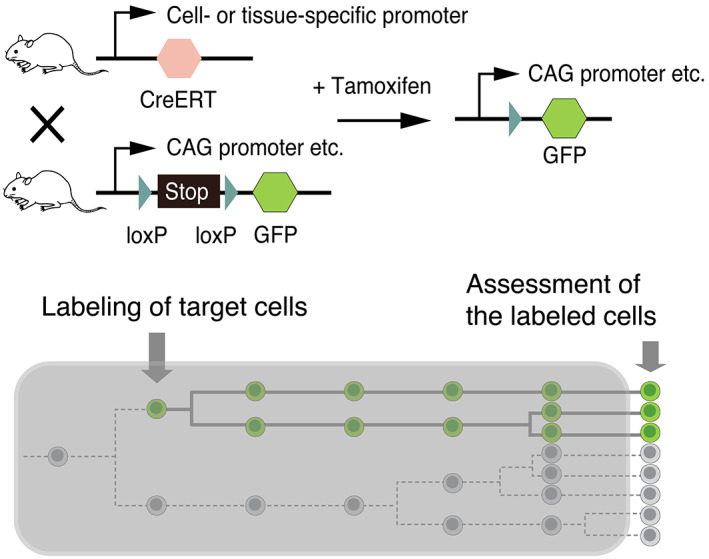
Schematics of the genetic lineage tracing strategy using the inducible Cre‐loxP system. Cre fused to a tamoxifen‐inducible mutated estrogen receptor (e.g., CreERT) is expressed under the control of a cell‐ or tissue‐specific gene promoter. In the presence of tamoxifen, Cre recombinase translocates to the nucleus and removes the Stop cassette flanked by two LoxP sites. After the temporally controlled excision of a Stop cassette, the reporter (e.g., GFP) is expressed in the target cells and all their progenies. Generally, from an experimental endpoint, the final distribution of labeled cells is assessed in fixed tissue specimens. Thus, the initial position of individual labeled cells and the behaviors of the labeled cells and unlabeled neighboring cells during the tracing period (shown in the gray box) are unknown

To identify the developmental origin of tissue stem cells, it is essential to obtain cell lineage data during development. Cell lineage tracing is an important technique that is widely used in developmental biology to acquire distribution information for the descendants of particular cells, and to evaluate the differentiation, mitotic potential, and migratory potential of cells (Kretzschmar & Watt, 2012). Since the early twentieth century, vital dyes such as neutral red, Nile blue, and PKH have been used extensively to label and trace cells of interest. Furthermore, vital dye labeling has been employed in analyses of neural crest cell migration, from the origin of the cells to the site of differentiation (Bronner‐Fraser & Fraser, 1988; Serbedzija et al., 1989). Since most long‐lived stem cells are considered to have a slow‐cycling and quiescent nature (Morris & Potten, 1994), a DNA label‐retaining technique that uses nucleoside analogs has also been used to identify and trace putative stem cells that divide infrequently as DNA label‐retaining cells (LRCs) (Barker et al., 2007; Buczacki et al., 2013; Cotsarelis et al., 1990; Tumbar et al., 2004). Furthermore, identification of the molecular markers of tissue stem cells has enabled the genetic labeling of specific stem cells using tissue‐specific gene promoters and site‐specific recombinase technology, such as the Cre‐loxP system (Figure 1). Genetic lineage tracing is now the standard experimental procedure used to assess tissue stem cell origins and cell lineage from development to adulthood. Moreover, unlike vital dyes, labeling with genetic markers does not spread to neighboring cells, and the labels are stably and permanently inherited by the progeny of the target cells (Kretzschmar & Watt, 2012; Soriano, 1999; Zinyk et al., 1998). As discussed below, cell lineage tracing, combined with other techniques such as live imaging, transcriptome analysis, in vitro assays, and theoretical approaches, has gradually exposed the origin of stem cells and the principles regarding the formation of adult stem cell populations in their final destinations, that is, stem cell niches. This review highlights studies on the hematopoietic system, brain, skeletal muscles, intestines, and hair follicles.

**FIGURE 2:**
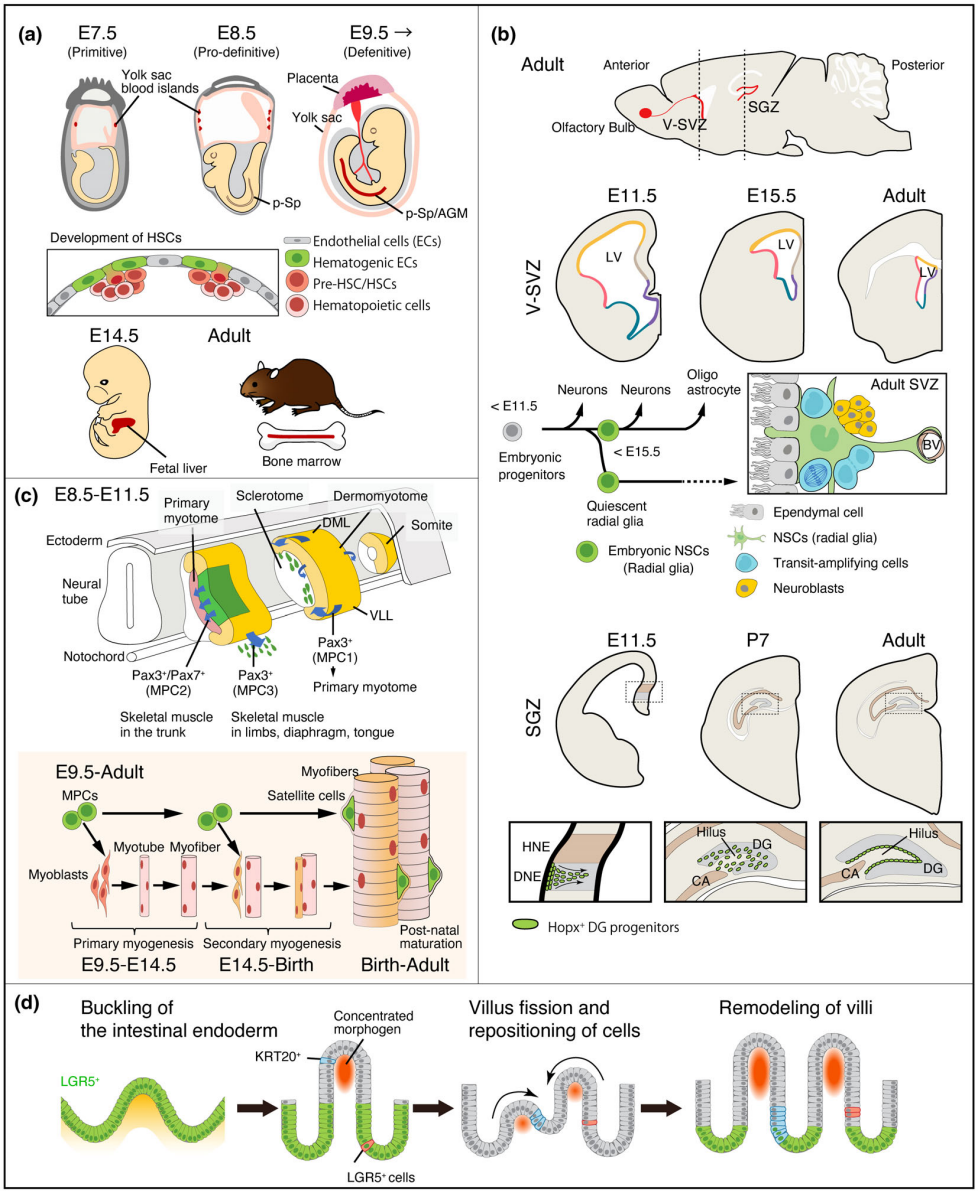
Recent findings regarding the developmental origin of tissue stem cells. (a) The development of hematopoiesis and hematopoietic stem cells (HSCs) in the mouse. Hematopoiesis emerges in three waves. The first and second hematopoiesis waves occur in the yolk sac blood islands at embryonic day (E) 7.5 and E8.5, respectively. The third wave commences on E9.5 with the HSCs/progenitors originating in both the yolk sac and the para‐aortic splanchnopleura (P‐Sp) and aorta–gonad–mesonephros (AGM) regions. The HSCs arise from a small population of endothelial cells. After E11.5, the HSCs gradually migrate and colonize the fetal liver and placenta before migrating home to the bone marrow just before birth. (b) Different models of neural stem cell (NSC) development in the ventricular–subventricular zone (V‐SVZ) and the subgranular zone (SGZ) in the mouse (modified from Cai & Yang, 2019). The development of V‐SVZ NSCs is explained by a “set‐aside” model. From the onset of V‐SVZ neurogenesis (E11.5), a precursor pool lining the dorsal (yellow line), dorsal lateral (red line), ventral lateral (blue line), and ventral medial (purple line) walls of the lateral ventricle (LV) initially generates cortical, striatal, globus pallidus, and septal neurons according to their locations. These precursor subsets of NSCs show the slowdown of cell‐cycle progression between E13.5 and E15.5, and NSCs are set aside in a quiescent state without participating in the generation of mature neurons, while other neural precursors continue to generate local neurons and glia. Adult dormant V‐SVZ NSCs in different regions produce specific sets of olfactory bulb interneurons after reactivation. Alternatively, a “continuous” model is proposed for the development of SGZ NSCs. Embryonic neural progenitors in the hippocampal neuroepithelium (HNE, beige) and the dentate neuroepithelium (DNE, gray) regions generate the cornu ammonis (CA, beige) and dentate gyrus (DG, gray) neurons, respectively. Hopx^+^ progenitors in the DNE continuously and exclusively contribute to dentate neurogenesis throughout development and adulthood. In the early postnatal stage, dentate NSCs gradually transition to quiescent NSCs, which disperse in the DG and hilus. BV, blood vessel. (c) The development of skeletal muscle and satellite cells in the mouse. All the skeletal muscles in vertebrates, except for certain head muscles, are derived from myogenic progenitor cells (MPCs, green) in the somite. The ventral compartment of the somite forms the mesenchymal sclerotome. The dorsal compartment remains epithelialized and forms the dermomyotome. MPCs, mainly from the dorsomedial (DML) and ventrolateral (VLL) lips of the dermomyotome (here they are termed as MPC1), delaminate to form the primary myotome. Subsequently, the central and ventrolateral parts of the dermomyotome generate the second and the third populations of myogenic progenitor cells (MPC2 and MPC3, respectively). After the MPCs reach their final destinations, they begin to differentiate to form primary myofibers. In vertebrates, skeletal muscle development principally occurs in two myogenic phases: (1) primary (embryonic) myogenesis, during which MPC‐derived myoblasts proliferate and fuse to form primary myofibers between E9.5 and E14.5, and (2) secondary (fetal) myogenesis in subsequent waves, during which additional fibers are generated along these template fibers. Secondary myogenesis is completed after birth, and results in most of the skeletal muscle fibers present at birth. During this phase, a subset of MPCs forms the pool of adult muscle stem cells—the satellite cells. The satellite cells attain a unique anatomical position between the plasmalemma and basement membrane of the adult myofibers. (d) The induction of intestinal stem cell coupling with intestinal villus morphogenesis during mouse development. Epithelial tissue buckling results in villi formation and leads to a local increase in morphogen in the mesenchyme and mesenchymal cell clustering beneath the tip of the villus. Concentrated mesenchymal signals instruct the LGR5‐positive (LGR5^+^) intestinal stem cell precursors to localize at the base of each villus. During the development of the gut, the villus undergoes major remodeling and fission. These structural changes cause the repositioning of the intestinal epithelial cells from the nonproliferative villus to the proliferative inter‐villus region and from the inter‐villus region to the villus. In a final wave of morphogenesis, the intestinal epithelial cells located at the base of the villi become adult intestinal stem cells

In mammals, successive waves of hematopoiesis occur at various anatomic sites during embryogenesis (Figure 2a) (Ghosn et al., 2019; Inlay et al., 2014). The initial wave arises in the yolk sac blood islands at embryonic day (E) 7.5 and generates primitive erythrocytes. After circulation is established at E8.5, the transient‐definitive hematopoietic wave, which generates erythro‐myeloid progenitors, begins in the yolk sac and placenta. After approximately E9.5, the final wave, known as the definitive hematopoietic wave, generates self‐renewing, multipotent hematopoietic stem cells (HSCs) that are capable of producing all blood cell types, including lymphocytes. The first self‐renewable and multipotent progenitors, known as pre‐HSCs, emerge in the yolk sac and the para‐aortic splanchnopleura (P‐Sp) and aorta–gonad–mesonephros (AGM) regions. At approximately E11, fully functional HSCs emerge in the AGM region and, subsequently, in the yolk sac and placenta. After E11.5, HSCs mainly colonize and expand in the fetal liver and placenta, and shortly before birth, they migrate from the fetal liver to the bone marrow, where they form a pool of adult HSCs that participate in hematopoiesis for the remainder of their lifespan.

Although extensive research exists on hematopoietic system ontogenesis, the origin of HSCs and their contribution to the embryonic and adult hematopoietic systems have been controversial owing to the difficulty in tracing the origin of a circulating cell population in the overlapping and transient hematopoietic waves (Ghosn et al., 2019; Neo et al., 2021; Weijts et al., 2021; Yoshimoto et al., 2008; Zovein et al., 2008). However, inducible lineage tracing methods that use the activities of different promoters have enabled the restricted labeling of subpopulations in each wave in the defined developmental time window, thus resulting in the accumulation of a vast amount of knowledge regarding the origin, development, and hematopoietic roles of HSCs (Neo et al., 2021). Furthermore, additional transcriptome analyses and transplantation assays have demonstrated that sequential interactions with distinct anatomical sites during development are important for the acquisition of stem cell properties (Copley & Eaves, 2013; Mikkola & Orkin, 2006; Weijts et al., 2021). This developmental history generates diversity in the hematopoietic potential of HSCs. It has also been shown that yolk sac progenitor cells, which were previously believed to be transient hematopoietic cells during embryogenesis, are involved in adult hematopoiesis (Inlay et al., 2014; Lee et al., 2016; Neo et al., 2021). Although these yolk‐sac‐derived hematopoietic progenitor cells were previously thought to be lineage restricted, they can differentiate into the mature blood cells of several adult hematopoietic lineages. Thus, the hematopoietic system is now considered to be much more diverse and flexible than the traditional hierarchical model posited, in which a single master stem cell produced a variety of differentiated cells responsible for the maintenance and regeneration of the hematopoietic system.

Two main populations of adult neural stem cells (NSCs) exist in the mammalian brain: NSCs in the ventricular–subventricular zone (V‐SVZ) of the lateral ventricles and NSCs in the subgranular zone (SGZ) of the hippocampal dentate gyrus (Figure 2b) (Cai & Yang, 2019). Until recently, no markers were available to specifically label the precursors of adult NSCs in the V‐SVZ. Thus, the DNA label‐retaining technique was employed to detect and fate map slow‐dividing cells during brain development (Fuentealba et al., 2015; Furutachi et al., 2015). These studies revealed that a subset of neural progenitor cells adopt a slow‐cycling/quiescent state during embryogenesis and become dormant adult V‐SVZ NSCs, while the other progenitors continue to generate neurons and glia during development. Alternatively, in the mouse dentate gyrus, lineage tracing using the Hopx‐CreERT2, which marks adult neural progenitors (Shin et al., 2015), showed that Hopx^+^ embryonic neural progenitors generate granule neurons throughout development and then transition into Hopx^+^ quiescent neural progenitors during an early postnatal period (Berg et al., 2019). Thus, two distinct models for the generation of NSCs have been proposed: a “set‐aside” model for generating adult V‐SVZ NSCs and a “continuous” model for generating adult SGZ NSCs. Additionally, more recent studies involving V‐SVZ NSCs combined single‐cell RNA‐sequencing (scRNA‐seq) with lineage tracing, and the results suggested that the transition from embryonic neural progenitor cells to dormant adult NSCs occurs from late embryogenesis to the first postnatal week, which is longer than previously thought (Borrett et al., 2020; Borrett et al., 2022). These and other studies have also suggested that NSCs are regionally specified and generate distinct region‐specific cell lineages, but that they share common signatures (Cai & Yang, 2019). Thus, further applications of novel technologies will provide data of unprecedented resolution and an improved understanding of the adult NSC generation process from embryonic progenitor cells.

Vertebrate skeletal muscle can regenerate, even after repeated acute injury. Satellite cells responsible for the regeneration of skeletal muscle were initially identified by Alexander Mauro as mononucleated cells, named for their anatomical “satellite” position between skeletal muscle fibers and the basement membrane (Mauro, 1961). Decades later, satellite cells were isolated using antibodies against cell surface markers, and subsequent cell transplantation showed that they are capable of self‐renewal and differentiation (Montarras et al., 2005; Sacco et al., 2008). Satellite cells in adult skeletal muscle generally exist in a quiescent state and are characterized through the expression of certain molecular markers (such as the paired box transcription factor Pax7) (Seale et al., 2000). Four studies that genetically ablated Pax7‐expressing satellite cells showed that skeletal muscle regeneration fails in the absence of satellite cells, establishing that the satellite cells are adult stem cells absolutely required for muscle regeneration (Lepper et al., 2011; McCarthy et al., 2011; Murphy et al., 2011; Sambasivan et al., 2011).

The results of several studies that involved the labeling of cells through genetic lineage tracing, electroporation, viruses, and quail‐chick transplantation suggested that the developmental origin of satellite cells is consistent with the origin of the muscle they reside in (Gros et al., 2005; Lepper & Fan, 2010; Relaix et al., 2005; Schienda et al., 2006). The myogenic stem/progenitor cell populations in embryos are classified into three subpopulations on the basis of their expressed genes and destinations (Figure 2c) (Tajbakhsh, 2009). In vertebrates, most skeletal muscles of the trunk and limbs are derived from somites. The dorsal part of the somite forms the dermomyotome (the origin of angiogenic, myogenic, dermal, and brown fat progenitors), and the ventral part of the somite forms the sclerotome (the origin of skeletal cells). The cells at the borders of the dermomyotome, mainly at the dorsomedial and ventrolateral lips, delaminate to form the primary myotome (which is the primitive skeletal muscle) in the medial somatic region (Gros et al., 2004; Relaix et al., 2005; Tajbakhsh et al., 1996; Tajbakhsh et al., 1997). These cells are the first myogenic progenitor cells expressing Pax3 (here they are termed MPC1), and, subsequently, once they reach their destination, they express the myogenic determination factors Myf5/Mrf4/(Myod). As the de‐epithelialization of the dermomyotome progresses, the proliferating myogenic progenitor cells that will prenatally form the muscle are released (Ben‐Yair & Kalcheim, 2005; Gros et al., 2005; Kassar‐Duchossoy et al., 2005; Relaix et al., 2005). These proliferating myogenic progenitors are the second and third myogenic progenitor cells (MPC2 and MPC3). MPC2 are released from the central dermomyotome into the underlying myotome, and they express Pax3/Pax7 (and subsequently Myod/[Myf5]). MPC3 migrate from the ventrolateral dermomyotome to form the skeletal muscles in the limbs, diaphragm, and tongue, and they express Pax3, Met, Lbx1, and Meox1 (and subsequently Myf5/Myod/[Mrf4]). MPC2 and MPC3 produce most of the juvenile satellite cells and, subsequently, adult satellite cells, whereas nondividing MPCs are proposed to be exhausted early in the embryo (Tajbakhsh, 2009).

Thus, it is widely accepted that satellite cells originate from dermomyotome‐derived MPCs. However, the process regarding the development of satellite cells from MPCs remains unclear. MPCs contribute to the successive phases of primary and secondary myogenesis (Figure 2c). In secondary myogenesis, a population of resident Pax3^+^/Pax7^+^ cells, which are muscle marker‐negative progenitor cells (lineage descendants of the dermomyotome), exists adjacent to the myofibers throughout the development and by the late fetal stages, is suggested to produce satellite cells. (Kassar‐Duchossoy et al., 2005). Other studies suggested that a great majority of the adult satellite cells is derived from the myogenic progenitors expressing Myf5 and MyoD during fetal stage (Biressi et al., 2013; Kanisicak et al., 2009; Wood et al., 2013). Moreover, recent studies revealed that fetal progenitors and juvenile and adult satellite cells are heterogeneous populations in terms of their gene expression, regenerative capacity, and self‐renewing ability (Rodriguez‐Outeirino et al., 2021). For example, a label‐retaining assay using TetO‐H2B‐GFP reporter demonstrated that 30% of satellite cells retained the H2B‐GFP label (LRCs) and were able to self‐renew, whereas the non‐LRCs were restricted to differentiation (Chakkalakal et al., 2014). Lineage tracing based on Myf5 expression showed that a 10% subpopulation of adult satellite cells had never expressed Myf5 and contributed to the satellite cell reservoir in vivo, while Myf5‐positive cells showed precocious differentiation (Kuang et al., 2007). It was also reported that adult satellite cells that show high expression of Pax7 are more undifferentiated than cells that show low expression of Pax7 (Rocheteau et al., 2012). Thus, further studies are required to clarify the origins and developmental processes that produce these “heterogeneous” adult satellite populations.

In the gut, adult epithelial stem cells reside in valley‐like crypts between the villi. During development, the villi are formed by the progressive folding of the epithelium and mesenchyme under the spatial constraints of smooth muscle (Shyer et al., 2013). Recent studies have demonstrated that the mechanical deformation of tissues during vilification creates a local signaling microenvironment that induces intestinal epithelial stem cells (Figure 2d) (Guiu et al., 2019; Shyer et al., 2015). Furthermore, a combination of biophysical modeling and lineage tracing using the expression of an adult intestinal epithelial stem cell marker (LGR5) indicated that embryonic intestinal epithelial cells are highly plastic and each embryonic epithelial cell has the potential to become a stem cell, regardless of stem cell marker expression (Figure 2d) (Guiu et al., 2019). Additional mechanistic insights are discussed below.

Genetic labeling using gene promoter activity has enabled the minimally invasive labeling of target cells in vivo for their long‐term lineage tracing and has contributed significantly to an understanding of the origin of tissue stem cells, as described above. However, this method has limitations. For example, in genetic cell lineage tracing, the specificity and efficacy of cell labeling are highly dependent on the cell‐type specificity and activity of the promoter used for the spatiotemporal control of Cre activity (Kretzschmar & Watt, 2012). Furthermore, the lineage of labeled cells is often statistically inferred on the basis of their initial and final distributions in fixed tissue specimens from multiple samples/animals without continuous observation in an identical sample. Generally, in fixed tissue specimens, the initial position of the individually labeled cells and their behavior during the tracing period are unknown (Figure 1). Therefore, the low specificity of cell labeling often results in difficulty in the interpretation of cell lineage tracing results and may lead to misinterpretations concerning the origin and behavior of the labeled cells. Specifically, certain conflicting claims concerning lineage analyses could, in part, result from the limitations of conventional genetical lineage tracing (Gonzales & Fuchs, 2017; Neo et al., 2021).

## 3 IDENTIFICATION OF THE ORIGIN AND LINEAGE OF HAIR FOLLICLE STEM CELLS

The mammalian hair follicle is a leading model for studying the molecular and cellular bases of tissue regeneration and stem cell regulation owing to its repetitive regeneration and degeneration cycles throughout life (Fujiwara et al., 2018; Xin et al., 2016). After the identification of LRCs in the hair follicle bulge region in 1990 (Cotsarelis et al., 1990), this model greatly enriched the understanding of the molecular characteristics, cellular dynamics, and physiological functions of adult stem cells and their niches, as well as their reciprocal interactions. At present, definitions are available for multiple stem cell compartments with clear boundaries and distinct functional contributions to hair follicle regeneration. However, the mechanisms by which stem cells are specified from seemingly homogeneous embryonic epithelial progenitors and compartmentalized in their niches during development remain unclear.

**FIGURE 3:**
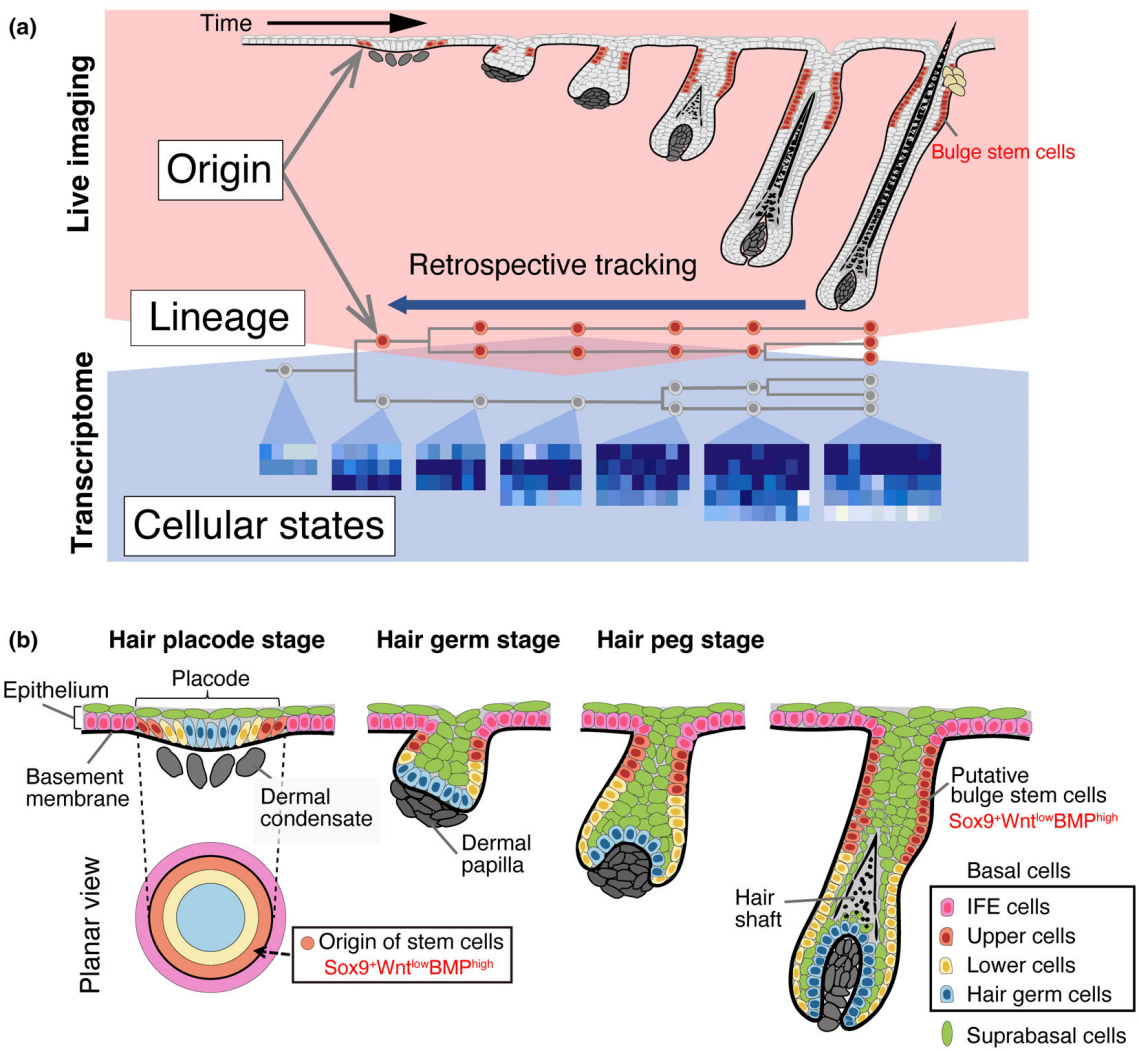
Recent findings on the developmental origin of hair follicle stem cells. (a) Strategy for elucidating the developmental origins of hair follicle stem cells. To obtain cell lineage information that directly links the developmental origin, lineage dynamics, and cell fate of each epithelial cell, three‐dimensional fluorescent live imaging of developing mouse hair follicles is performed. By continuously and retrospectively tracking the hair follicle epithelial cells via video, detailed lineage information is obtained. In parallel, time‐course single‐cell transcriptome analysis is performed to profile the cellular states. The integration of data from both analyses reveals where and how epithelial cell populations, including prospective bulge stem cells, are progressively generated and diversified. (b) The developmental origin of hair follicle bulge stem cells and a new model for hair follicle development: the telescope model. In mouse hair follicle development, concentric ring‐shaped cell prepatterns on the flat epithelium of the hair placode transform into longitudinally aligned cylindrical compartments through their extendable telescope‐like expansion. The putative hair follicle bulge stem cells originate from the peripheral ring zone of the hair placode and develop in the compartment displaying a Sox9‐positive/Wnt‐signal‐activity‐low/BMP‐signal‐activity‐high state (Sox9^+^Wnt^low^BMP^high^) throughout development. IFE, interfollicular epidermis

Hair follicle development is initiated by the formation of a placode, which is a localized thickening in the embryonic epithelial sheet overlying the dermal condensates. The placode, which produces all the cells in adult hair follicles, invaginates toward the dermis and develops into the hair germ, hair peg, and bulbous peg, which is accompanied by the development of hair follicle stem cells (Figure 3) (Millar, 2002). A previous study reported that hair follicle stem cells are derived from Sox9‐positive/Wnt‐signal‐activity‐low suprabasal cells produced by the perpendicular and asymmetric cell division of placode basal cells (Ouspenskaia et al., 2016). When Sox9‐expressing cells were labeled with Sox9‐CreERT2 at the placode stage, the labeled cells were detected in the suprabasal cells of the hair placode. After the chase period, the labeled cells were detected in a large area of hair follicles, including the hair follicle stem cell niche, that is, the bulge. Sox9 is an important transcription factor of adult hair follicle stem cells that is required for stem cell specification and is known to be expressed in the hair placode with other hair follicle stem cell markers such as Lhx2 (Nowak et al., 2008; Rhee et al., 2006). However, as the expression patterns of these adult stem cell markers have been shown to differ in the placode, it is unclear whether Sox9 could serve as an early stem cell marker to specifically distinguish the precursors of stem cells from other differentiating epithelial precursors (Nowak et al., 2008; Rhee et al., 2006; Xu et al., 2015).

A recent study revisited the developmental origin of hair follicle stem cells by combining two comprehensive and unbiased single‐cell methods: lineage analysis using the long‐term, continuous, live imaging of hair follicle development and time‐course single‐cell transcriptomes (Figure 3a) (Morita et al., 2021). In this study, a live imaging method for entire developing hair follicles was established using an ex vivo culture of embryonic whisker and dorsal skin tissues (Morita et al., 2016; Morita et al., 2021). This method enables the continuous visualization of the cellular dynamics at single‐cell resolution without relying on specific molecular markers, from the placode formation stage to the later developmental stages during which basal epithelial cell compartmentalization, cellular differentiation, and hair shaft formation occur. By tracing hair follicle epithelial cells within identical hair follicles, it is possible to obtain four‐dimensional (4D) single‐cell lineage data linked to information on the cell position and division within the tissue, as well as the topological relationships among the cells. This cell lineage data provide a dynamic 4D cell lineage atlas that directly connects the cell origin, cellular dynamics, and final distribution of each cell. Moreover, this cannot be obtained by conventional genetic lineage tracing using the histological detection of labeled cells in fixed tissues. Using this type of analysis, the authors of the above‐mentioned study demonstrated that the precursors of different epithelial lineages are aligned in a two‐dimensional (2D) concentric manner in the basal layer of the placode. As development progresses, each concentric ring region in the placode invaginates toward the mesenchyme and elongates longitudinally so that individual ring regions develop into distinct three‐dimensional (3D) cylindrical compartments, including the bulge stem cell compartment (Figure 3b). Moreover, the putative hair follicle stem cells originate from a Sox9‐positive peripheral ring zone of placode basal cells but not from suprabasal Sox9‐positive cells, as was previously suggested. To reconcile the apparent discrepancy in the origin of hair follicle stem cells, the fate of individual Sox9‐positive placode cells was examined using a live imaging system. The results showed that Sox9‐positive cells exist in the suprabasal layer of the placode, as well as in the basal layer of the placode periphery, and that only Sox9‐positive basal cells produce future stem cells.

Oriented cell division often determines the fate of daughter cells by providing either intrinsic or extrinsic differences: intrinsically, cytoplasmic cell fate determinants are asymmetrically segregated in daughter cells; extrinsically, two daughter cells are placed in different microenvironments (Santoro et al., 2016). To analyze the underlying cell fate choice mechanisms in the placode, Morita et al. (2021) examined the relationship between the angle of the cell division orientation and the fate of the daughter cells using live imaging data. The authors observed that, irrespective of the angle of cell division, most of the daughter cells from the placode basal layer in each concentric zone contribute to the basal cells of their future cylindrical compartment. Specifically, both the upper and lower daughter cells of the perpendicular division in the prospective stem cell region contribute significantly to the future stem cell region. These results indicate that the fate of the placode basal cells is determined by the cell position rather than the cell division orientation.

To explain the spatiotemporal transcriptional changes in each cell lineage throughout the hair follicle development, Morita et al. (2021) obtained time‐course single‐cell transcriptome data for the entire epithelium of developing hair follicles. Reconstruction of the tissue spatial arrangement of the placode cells revealed that there was a concentric ring‐shaped gene expression pattern in the placode, which was consistent with the cell lineage fate map pattern obtained through live imaging. The peripheral ring zone of this concentric pattern, in which the stem cell precursors originate, displayed a Sox9‐positive/Wnt‐signal‐activity‐low/BMP‐signal‐activity‐high state, which are the characteristics of adult bulge stem cells. The reconstruction of the developmental trajectories enabled the identification of five major epithelial cell lineages that diverge just after the follicle invagination stage and develop into the mature later compartments as an independent lineage; this was consistent with the direct cell lineage tracking data obtained from the live imaging. Within the stem cell lineage, several stem cell markers were upregulated throughout development in a low Wnt‐signaling state and high BMP‐signaling state, which was the same cellular state as the adult stem cells. This suggests that the creation, development, and maintenance of this lineage and its microenvironment may be achieved by organizing a compartmentalized local signaling zone during hair follicle development.

## 4 THE “TELESCOPE MODEL” UNDERLIES HAIR FOLLICLE COMPARTMENTALIZATION AND STEM CELL FORMATION

The 4D dynamic atlas of hair follicle development revealed intriguing coordination of morphogenesis and cell fate specification, which is common in various types of hair follicles: The concentric ring‐shaped prepattern of cell lineage and gene expression is transformed into longitudinally aligned, cylindrical compartments as the hair follicle invaginates and elongates (Figure 3b) (Morita et al., 2021). This developmental patterning of the hair follicle is named the “telescope model” in analogy to the extension of an expandable telescope. The formation of 3D cylindrical compartments from 2D concentric ring domains resembles the mechanism of leg primordium development in *Drosophila* embryogenesis, suggesting that this may be a universal morphogenetic patterning process in organogenesis throughout many organs and species (Lecuit & Cohen, 1997; Ruiz‐Losada et al., 2018). Furthermore, for the development of functional organs and their subsequent maintenance, a variety of specialized cells, including tissue stem cells, must be appropriately specified and arranged during organogenesis. The telescope model may explain the spatiotemporal coordination of tissue morphogenesis and cell fate specification during the formation of 3D cylindrical compartments consisting of various epithelial cell lineages, one of which is a stem cell lineage.

This patterning mechanism also indicates that, unlike adult hair follicle stem cells, putative hair follicle stem cells do not actively contribute to the formation of the lower part of the hair follicle and hair shaft; they contribute only to the development of the stem cell region during development. This suggests that hair follicles adopt a strategy of forming and setting aside putative stem cells position dependently at an early developmental stage and acquiring their properties and quiescent state gradually.

To date, the mechanism underlying the establishment of concentric zones has not been elucidated. Wnt/β‐catenin signaling is activated in the prospective placode region and is required for placode formation (Saxena et al., 2019). Interestingly, a small‐patched forced activation of Wnt/β‐catenin signaling in a uniform, flat, embryonic epidermis induced Sox9 in the surrounding cells (Ouspenskaia et al., 2016), suggesting that Wnt signal‐active epithelial cells at the center of the placode may induce the origin of stem cells. Because the epidermal Wnt/β‐catenin signaling triggers many critical epithelial–mesenchymal signaling interactions during hair follicle development (Qu et al., 2022; Saxena et al., 2019), the interplay between the placode epithelium and underlying mesenchyme may also play a critical role in this patterning process.

## 5 THE COORDINATION OF MORPHOGENESIS AND STEM CELL FORMATION

It is important to understand how the behavior and fate of individual cells (including stem cells), as well as tissue‐scale patterning and morphogenesis, are appropriately coordinated with spatial and temporal precision, not only in embryonic organogenesis but also in adult tissue maintenance and regeneration. Recent studies have highlighted the importance of tissue architecture in controlling the fate of individual cells.

For adult organs, the contributions of the structure and organization of tissue in stem cell regulation have been reported (reviewed in Goodell et al., 2015; Xin et al., 2016). In skin, hair follicles, intestines, and the hematopoietic system, the tissue stem cell pool is composed of heterogeneous cell populations, which hold different transcriptional profiles and contribute only to the differentiation program for their territory. However, upon tissue injury, these stem cells display a high degree of plasticity to complement each other and repair tissues beyond their original role (Xin et al., 2016). For example, in the hair follicle, bulge stem cell or hair germ cell ablation via a laser induces the repopulation of epithelial cells from neighboring compartments and their proceeding acquisition of the identities of lost stem cells (Rompolas et al., 2013). However, when the epithelium is slightly physically separated from the mesenchyme, resulting from laser ablation, the full recovery of the stem cells is impaired, even if the mesenchyme remains just below the hair follicle. This implies that the fate and behaviors of stem cells are plastic but, ultimately, they are governed by the precise tissue organization of the niche, such as the cells, extracellular matrix, and other noncellular material (Koester et al., 2021; Xin et al., 2016).

Recently, a close relationship between stem cell formation, niche formation, and tissue topology has been reported in developing mouse and chicken intestines (Guiu et al., 2019; Shyer et al., 2015). In the early stages of intestinal development, embryonic progenitors that express the stem cell marker LGR5 are uniformly distributed throughout the flat epithelium (Figure 2d). Shyer et al. (2015) showed that, when the villi are formed by the buckling of the intestinal endoderm, sonic hedgehog (Shh) signaling, which is uniformly expressed throughout the intestinal endoderm, is increased locally at the curved tip of the folded epithelium, leading to the induction of a signaling center in the adjacent mesenchyme. Furthermore, bone morphogenic proteins (BMPs) secreted from this signaling center are fed back to the apical epithelium to antagonize Wnt activation, inhibiting the proliferation of the Wnt‐dependent LGR5‐positive cell population and, consequently, confining the stem cells to the base of each villus. Thus, the mechanical deformation of the tissue from 2D to 3D creates local signaling centers in uniform morphogenetic fields and triggers the local induction and differentiation of cells (Shyer et al., 2015). Additionally, using 3D imaging and quantitative lineage tracing, Guiu et al. (2019) demonstrated that mouse fetal villi undergo remodeling and fission as tissue grows and that tissue remodeling allows nonproliferative LGR5‐negative/KRT20‐positive villus cells to migrate into proliferative regions to contribute to adult stem cells. Moreover, biophysical modeling revealed that the observed long‐term equipotency of developing epithelial cells cannot be explained by a model based on a unidirectional migration of cells from the gut base to the villi, as in the adult intestine. However, this can be explained by a model in which cell repositioning occurs between the villi and inter‐villi regions as the villus undergoes remodeling and fission (the cell‐repositioning model). These results indicate that, regardless of the location or marker expression, embryonic intestinal cells are generally highly plastic, and all cells are equivalent precursors of adult intestinal stem cells, suggesting that the subsequent cell fate can be determined position dependently (Guiu et al., 2019). Thus, changes in tissue topology, such as tissue buckling and villi fission during intestinal morphogenesis, provide a biophysical mechanism to modify the local microenvironment, allowing for the reconciliation of morphogenesis and cell fate. Although it is unclear whether this applies to the developmental processes of other organs and tissues, such as hair follicles, it is assumed that organ‐specific morphogenetic processes influence stem cell induction, given the increasing evidence that tissue geometry and mechanical stimuli to cells contribute to cell proliferation, survival, and cell fate determination (Chan et al., 2017; Gjorevski et al., 2022). Thus, understanding the formation of tissue stem cells and their niches during development requires comprehension of the molecular, cellular, and mechanical systems that act across varying scales (genetic, cellular, multicellular, and tissue) through tissue architecture and organization.

## 6 CONCLUSIONS AND PERSPECTIVES

In this review, we highlighted the coordination between stem cell formation and developmental tissue patterning, which recent emerging technologies have revealed. Studies on the intestinal epithelium and hair follicle suggest that stem cell properties are induced position dependently. In the intestine, all embryonic progenitors can become adult stem cells, but only cells positioned in the crypts become stem cells (Guiu et al., 2019; Shyer et al., 2015). Furthermore, the positioning of progenitors in the crypts is determined via mechanical tissue buckling, which forms a signaling zone similar to the adult intestinal stem cell niche. Similarly, in the hair follicle, the stem cells are derived from the ring‐shaped signaling zone, which in part exhibits the adult stem cell state, in a planar skin morphogenetic field (Morita et al., 2021). In the skin, however, there are no definitive geometrical tissue changes before the formation of the concentric prepattern (Saxena et al., 2019), while dermal cell aggregation beneath the placode is a key change in tissue organization concomitant with the formation of concentric zones. Thus, it would be interesting to investigate how concentric prepattern formation, dermal cellular aggregation, and the subsequent tissue invagination contribute to the emergence and development of tissue stem cells.

After the emergence of stem cell precursors, maturation toward adult stem cells is observed in many organs. Additionally, quiescence is a prominent characteristic of adult stem cells, which is conferred during the embryonic and perinatal development of the hair follicle, skeletal muscle, and brain (Berg et al., 2019; Fuentealba et al., 2015; Furutachi et al., 2015; Nowak et al., 2008; Tajbakhsh, 2009). In hair follicles, once the bulge stem cell precursors are formed in the peripheral ring zone of the placode, they mature into pre‐bulge cells as a linear lineage, even if the hair follicle shows dynamic morphological changes (Morita et al., 2021). Moreover, the core transcriptional and positional identity of NSCs does not change from the active embryonic state to the quiescent adult state (Borrett et al., 2020; Borrett et al., 2022). These findings suggest that the process of the generation of adult tissue stem cells, including the acquisition of the quiescent state, may proceed gradually as a single linear lineage within a compartmentalized signaling domain. Furthermore, the state of the signaling domains may change in tandem with hierarchal regional specification and tissue shaping during morphogenesis. Thus, in hair follicles, the telescope model could provide a framework for examining the coordination of tissue morphogenesis, signal zone organization, and lineage development.

The present challenge is to decipher the causal links between tissue patterning/shaping and cell fate specification over multiple biological scales. Recent studies have indicated that changes in tissue morphology and the cellular state are coupled via the feedback between mechanical and biochemical patterning events (Hannezo & Heisenberg, 2019). Emerging technologies, such as live imaging, single‐cell sequencing, spatial transcriptomics, barcoding‐based lineage tracing, and mechanical force measurement, will aid in obtaining multi‐omics information over various biological scales. These technologies will also provide more detailed and accurate information regarding when and how stem cells establish their own identities and mature into the adult stem cells responsible for maintaining adult homeostasis. To utilize these technologies, it is crucial to understand the advantages and disadvantages of each method. For example, live imaging can significantly increase the spatiotemporal resolution and continuity of information concerning the dynamics of cells and signals, but each biological model and question requires a specific imaging setting. Long‐term and high‐throughput analysis is also challenging in most live‐imaging experiments. In contrast, single‐cell transcriptomes enable high‐throughput profiling of vast numbers of cells, but the cells lose information regarding the spatial and temporal organization. Spatial transcriptomics allows one to profile cellular states using spatial information in the *XY* plane, but not in three dimensions. As described at the end of the second section of this review, genetic lineage tracing also has limitations; however, it remains a powerful tool. Thus, conceptual morphogenetic frameworks and multifaceted analyses that combine the advantages of each method will lead to an improved understanding of the principles underlying the construction of the tissue stem cell system.
